# Exploring the dynamics of prefrontal cortex in the interaction between orienteering experience and cognitive performance by fNIRS

**DOI:** 10.1038/s41598-024-65747-1

**Published:** 2024-06-28

**Authors:** Jingru Liu, Yang Liu, Linzhen Wu

**Affiliations:** 1 Department of Physical Education, Xi’an University of Posts and Telecommunications, Xi’an, 710199 Shaanxi China; 2 School of Physical Education, Shaanxi Normal University, Xi’an, 710121 Shaanxi China

**Keywords:** Orienteering, Spatial memory, Mental rotation, Near-infrared functional brain imaging (fNIRS), Prefrontal cortex dynamics, Cognitive neuroscience, Human behaviour

## Abstract

Sporting experience plays a pivotal role in shaping exercise habits, with a mutually reinforcing relationship that enhances cognitive performance. The acknowledged plasticity of cognition driven by sports necessitates a comprehensive examination. Hence, this study delves into the dynamic intricacies of the prefrontal cortex, exploring the impact of orienteering experience on cognitive performance. Our findings contribute empirical evidence regarding the functional activation of specific brain regions bridging the nexus between experiential factors and cognitive capabilities. In this cross-sectional study, a cohort of forty-nine athletes was enrolled to meticulously examine behavioral variances and prefrontal cortex dynamics among orienteering athletes of varying experience levels across diverse non-specialized scenarios. These investigations involved the utilization of functional near-infrared spectroscopy (fNIRS) to detect alterations in oxygenated hemoglobin (HbO2). The high-experience expert group exhibited neurological efficiency, demonstrating significantly diminished brain activation in the dorsolateral prefrontal, left ventral lateral prefrontal, and right orbitofrontal regions compared to the low-experience group. Within the low-experience novice group, superior performance in the spatial memory task was observed compared to the mental rotation task, with consistently lower reaction times across all conditions compared to the high-experience group. Notably, cerebral blood oxygenation activation exhibited a significant reduction in the high-experience expert group compared to the low-experience novice group, irrespective of task type. The dorsolateral prefrontal lobe exhibited activation upon task onset, irrespective of experience level. Correct rates in the spatial memory task were consistently higher than those in the mental rotation task, while brain region activation was significantly greater during the mental rotation task than the spatial memory task.” This study elucidates disparities in prefrontal cortex dynamics between highly seasoned experts and neophyte novices, showcasing a cognitive edge within the highly experienced cohort and a spatial memory advantage in the inexperienced group. Our findings contribute to the comprehension of the neural mechanisms that underlie the observed cognitive advantage and provide insights into the forebrain resources mobilized by orienteering experience during spatial cognitive tasks.”

## Introduction

Wayfinding and navigation is a survival skill necessary for human beings over the years of evolution. Intelligent and fully automated navigation in modern life replaces the human brain in processing information^1^. However, the prolonged adherence to predetermined trajectories, devoid of active cognitive engagement, has been observed to compromise certain brain functions. This, in turn, fosters an increased reliance on external cues, culminating in a decline in spatial processing abilities and the consequential atrophy of associated neural structures^2–4^.

Orienteering, a sport often likened to a strategic chess game in motion^5^, stands out as a highly integrated physical and intellectual pursuit. In this discipline, athletes adeptly employ compasses and maps to navigate unfamiliar terrain, necessitating swift map interpretation for effective wayfinding. Experienced orienteers demonstrate a remarkable ability to seamlessly transition between various cognitive tasks, showcasing mental agility and efficiency^6^.

Quoting Thomas Edison’s famous assertion that “Genius is one percent inspiration, ninety-nine percent perspiration,” underscores the argument that consistent skill practice over weeks contributes to athletic improvement. This sentiment aligns with evidence from studies highlighting the intricate involvement of the brain’s nervous system, revealing a positive correlation between motor experience and skill proficiency^7^. This study delves into the neural underpinnings of such correlations, shedding light on the impact of orienteering experience on cognitive and spatial abilities.

Sports experience and cognitive ability are intertwined, and together they shape an individual’s behavior and thinking patterns. Motor experience is not only a simple accumulation of body muscle movement, but also includes the individual’s cognitive ability complementary to the perception of environmental changes, the execution of the action and the feedback of the result^8–15^. It has become an established fact that exercise can effectively promote cognition. According to Ericsson et al.^16^, it takes at least 10 years or 10,000 hours of intensive practice to become an expert in a particular sport discipline. Sport experience is one of the core elements that create sport experts, and this is also true for orienteering. Psychology places EF (inhibition, working memory and cognitive flexibility) at the center of cognitive assessment^17,18^, and they are the basis for the brain’s ability to process higher-order thinking such as “reasoning, decision-making, and planning”. The need for orienteers to apply cognitive abilities to navigate, make decisions, and make judgments in unfamiliar environments confirms that sport experience is closely related to cognitive functioning. For example, orienteers make quick decisions about routes in environments with varied terrain, landforms, and vegetation (cognitive flexibility), reading map information, and making cognitive judgments in the brain (spatial memory, mental rotation). Therefore, both spatial memory and mental rotation are a kind of cognitive ability^19^. Relevant studies have pointed out that expert orienteering athletes show a certain advantage in spatial memory ability in specialized scenarios^20,21^, caused by the reasons mainly stemming from the perennial sports experience and specialized training^22^. It has also been pointed out that orienteering athletes’ mental rotation ability constrains the efficiency of map literacy, and that sports experience can influence the development of cognitive abilities by changing the structure and function of the brain^16^.

With the profound advancements in cognitive neuroscience, there is a wealth of technical resources and conceptual frameworks available for investigating cognitive mechanisms in orienteering athletes. Functional near-infrared spectroscopy (fNIRS), noted for its resilience against head movements, safety, portability, high temporal and spatial resolution, and ability for prolonged and repeated measurements^23,24^, has been extensively employed in the study of various sports, such as soccer^25^, badminton^26^, and tai chi^27^. Functional near-infrared spectroscopy (fNIRS) has become an important tool for studying spatial working memory. Studies have shown that fNIRS can effectively monitor cortical haemodynamics during spatial memory tasks^28^. The prefrontal lobes are significantly activated during spatial working memory tasks^29^. The dorsolateral prefrontal cortex (DLPFC), among others, has been identified as a potential brain target for fNIRS neurofeedback to enhance spatial memory in humans^30^. The fNIRS neurofeedback has been shown to enhance spatial memory in humans.This technology has not only established a scientific foundation for understanding the cognitive processing mechanisms of the brain but has also provided indispensable technical support for our present study.

The experience of sport is fundamental in shaping brain activity and neuroplasticity.Numerous studies have underscored the significance of the prefrontal cortex (PFC) in various cognitive functions, encompassing working memory, decision-making, executive functions, and planning processes^31–33^. This key region orchestrates higher cognitive functions and behaviors, with specific sub-regions like the ventral prefrontal cortex (VLPFC) and dorsal prefrontal cortex (DLPFC) exhibiting heightened activity during working memory tasks^34^. The influence of prior experience on neural processes is evident in the modulation of brain activity observed by Kraeutner et al.^35^ in a sport cognition-related task. The dorsomedial prefrontal cortex has been implicated in supporting spontaneous thought experiences^36^. Additionally, the prefrontal cortex collaborates with other brain regions, such as the hippocampus, during spatial memory tasks^37–39^. Together, these findings highlight the complex relationship between experience and brain activity. They highlight the importance of motor experience in shaping brain activity to improve neural efficiency and promote sports skills. How sports experience affects neural processes and cerebral hemispheric function is further elaborated.

Interestingly, previous research has overlooked variations in brain region activity among orienteering athletes with different experience levels. Therefore, understanding which specific prefrontal cortex regions are activated and to what extent during different spatial tasks in orienteering athletes of varying experience levels has become a novel focal point for investigating prefrontal cortex dynamics.

In this study, we designated the prefrontal cortex (PFC) as the primary region of interest and devised a non-specific cognitive task centered on visual information processing. By focusing on the cognitive tasks within non-specific scenarios, we delved into the behavioral performance and neural activity basis of the prefrontal cortex in highly experienced expert orienteers and low-experience novices. Two spatial cognitive indices, namely spatial memory and mental rotation were employed to explore the intricate relationship between behavioral performance and brain neural activity in orienteering athletes with distinct experience levels. This exploration aimed to unveil the intrinsic mechanisms behind the cognitive advantage exhibited by highly experienced orienteers, offering insights into the inherent associations of cognitive processing characteristics within non-specialized scenarios in orienteering sports at varying experience levels. Our objective is to elucidate the interplay between experience and prefrontal cortex dynamics within the cognitive neural network, providing conceptual foundations for optimizing neural network structures.

## Results

### Behavioral performance

#### Behavioral performance of spatial cognition in non-specialized scenarios in orienteering athletes of different levels

A 2 (group: high experience-expert group, low experience novice group) * 2 (task type: spatial memory, mental rotation) two-factor repeated-measures ANOVA was employed to statistically scrutinize the correctness rate of spatial cognition tasks in non-specialized scenarios. This analysis aimed to test the main effect and interaction effect of exercise level on different spatial cognition tasks. Additionally, an independent samples t-test was utilized to examine group differences in response time across different tasks. Descriptive statistics results are presented in Table 1.

**Table 1:** List of behavioral results of spatial cognitive tasks in non-specific scenarios (M±SD)).

Group	Correctness (%)		Response time (ms)	
	Spatial memory	Mental rotation	Spatial memory	Mental rotation
High-Experienced Expert Group	0.79 ± 0.07	0.81 ± 0.04	1038.46 ± 165.77	3208.78 ± 440.56
Low-experience Novice group	0.76 ± 0.06	0.68 ± 0.05	1143.25 ± 127.86	3770.14 ± 315.08

1. Correctness Results: The analysis revealed a significant main effect for group $[{F}(1,47)=35.709,{p}=0.001>0.05,{~\eta p}2=0.452]$, indicating that the high experience-expert group exhibited a significantly higher correctness rate compared to the low experience-novice group. A significant main effect for task type was also observed $[{F}(1,47)=5.332,{p}=0.025>0.05,{~\eta p}2=0.102]$, with the spatial memory task displaying a notably higher correctness rate than the mental rotation task. Furthermore, a significant interaction effect between group and task type emerged $[{F}(1,47)=16.287,{p}=0.001>0.05,{~\eta p}2=0.257].$

**Figure 1:**
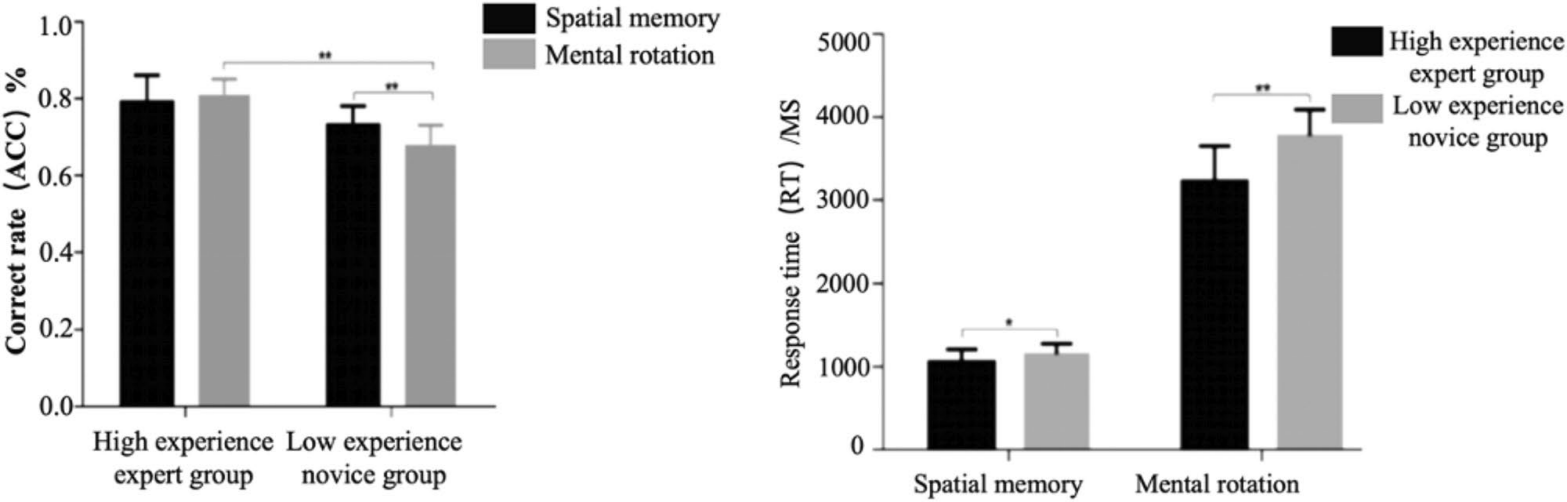
Behavioral results of spatial cognitive tasks in non-specialized scenarios for orienteering athletes of different levels $$(Note: * represents 0.001<p<{0.05}** represents p\le 0.01)$$.

A subsequent simple effects test unveiled a nonsignificant group main effect in the spatial memory task condition ${F}(1,47)=2.290,{p}=0.137>0.05,\mathrm {~\eta p}2=0.046]$, indicating no significant difference between experts and novices. However, a significant group main effect was observed in the mental rotation task $[{F}(1,47)=70.277,{p}=0.001>0.05,\mathrm {~\eta p}2=0.559]$, with the high experience-expert group exhibiting significantly higher correctness than the low experience-novice group. In the low experience-novice group condition, the spatial memory task demonstrated significantly higher correctness than the mental rotation task ${F}(1,47)=29.008,{p}=0.001>0.05,\mathrm {~\eta p}2=0.382]$. Conversely, in the high experience-expert group condition, there was no statistical difference between spatial memory and mental rotation $[{F}(1,47)=1.144,{p}=0.291>0.05,\mathrm {~\eta p}2=0.024]$. Shown in Fig. 1(left). This observation may stem from the effective improvement of both spatial memory and mental rotation after prolonged orienteering practice in the high-experience-expert group.

2. Reaction-Time Results: An independent samples t-test was performed on the mean reaction time of the two groups in different spatial tasks. The results indicated a significantly lower reaction time in the high experience-expert group compared to the low experience-novice group under both spatial memory and mental rotation tasks (t = − 2.461, p = 0.018 < 0.05, 95% CI: − 190.479 to − 19.1178; t = − 5.157, p = 0.001 < 0.05, 95% CI: − 780.343 to − 342.366) Fig. 1(right), suggesting faster spatial cognitive processing in the high-experience-expert group.

#### fNIRS results of different levels of orienteering athletes in non-specialized scene tasks

To investigate the neural activity in the prefrontal lobe (PFC) among orienteering athletes at different experience levels during spatial cognitive tasks in non-specialized scenarios, a 2 (group: high experience-expert, low experience-novice) $\times$2 (task type: spatial memory, mental rotation) two-factor repeated-measures ANOVA was conducted. Task and group served as independent variables, with oxygenated hemoglobin HbO2 ($\beta$-value) in each channel of the prefrontal lobe as the outcome variable.

**Figure 2:**
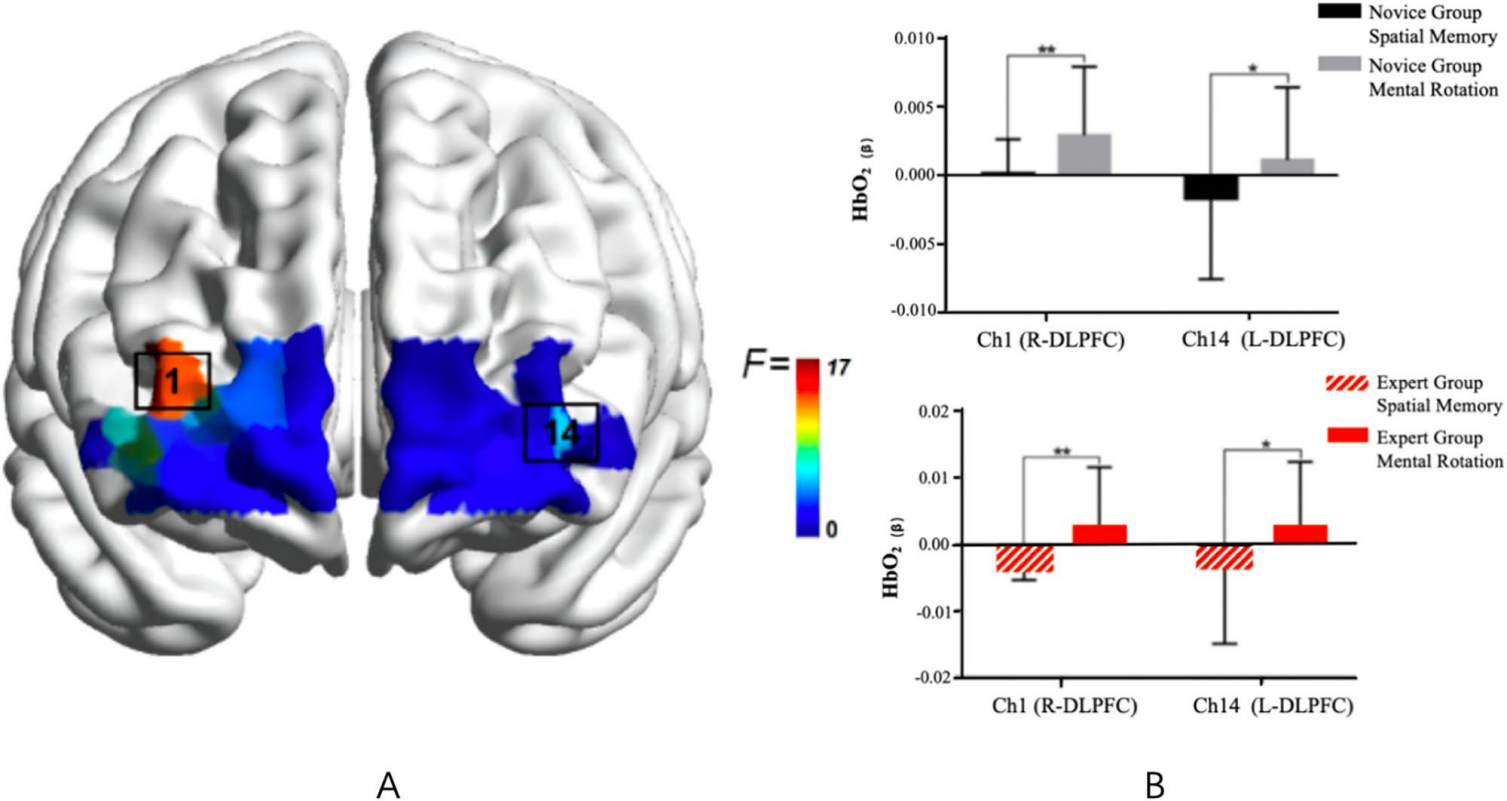
Task main effect F-value activation plots (left) and task differences under significant channels (right) $$(Note: * represents 0.001<p<{0.05}** represents p\le 0.01)$$.

The results revealed significant main effects of task type in specific channels: Ch1 (R-DLPFC)${F}(1,47) = 17.697,{p}=0.000>0.05,\mathrm {~\eta p}2=0.273]$ and Ch14 (L-DLPFC) ${F}(1,47) = 5.489,{p}=0.023>0.05,\mathrm {~\eta p}2=0.105]$ . Under the mental rotation task, cerebral blood oxygen activation in these channels was significantly higher than during the spatial memory task for both groups of subjects. No other channels exhibited statistical differences under either task condition Fig. 2.

**Figure 3:**
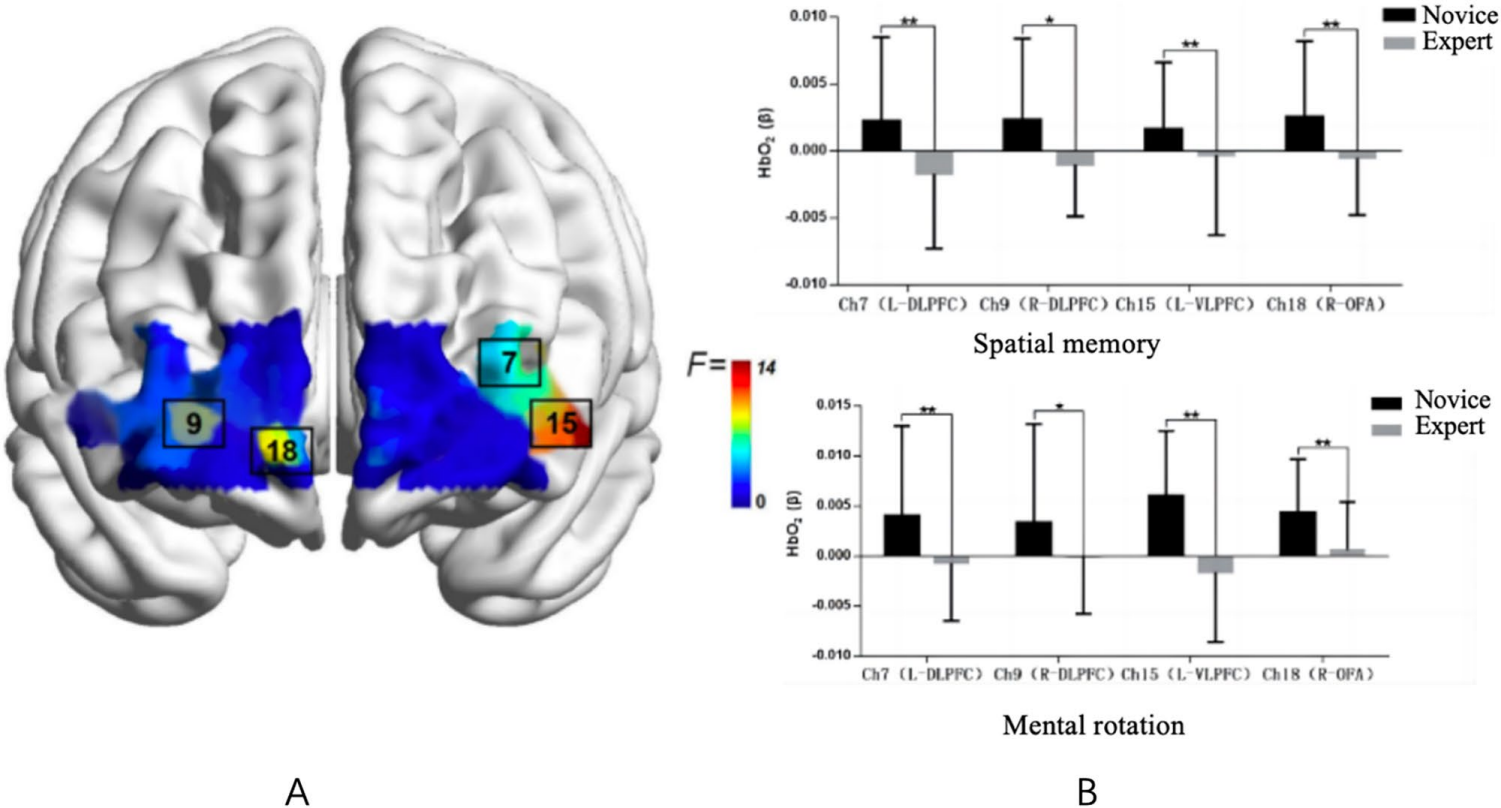
Activation plots of F-values for group main effects (left) and group differences under significant channels (right)$$(Note: * represents 0.001<p<{0.05}** represents p\le 0.01)$$.

Channels with noteworthy group main effects were identified, including Ch7 (L-DLPFC) ${F}(1,47)=7.738,{p}=0.008>0.05,\mathrm {~\eta p}2=0.141]$, Ch9 (R-DLPFC) ${F}(1,47)=5.948,{p}=0.019>0.05,\mathrm {~\eta p}2=0.112]$, Ch15 (L-VLPFC) ${F}(1,47)=13.937,{p}=0.001>0.05,\mathrm {~\eta p}2=0.229]$, and Ch18 (R-OFA) ${F}(1,47)=8.883,{p}=0.005>0.05,\mathrm {~\eta p}2=0.1590]$. In both task conditions, cerebral oxygenation activation in these channels was significantly lower in the expert group compared to the novice group Fig. 3.

No significant interactions were observed across all channels based on task type and group, and therefore, further analysis of these interactions was not pursued. Summarizing the study results, task type induced variations in cerebral blood oxygen activation in the dorsolateral prefrontal lobe (DLPFC), with significantly higher activation during the mental rotation task compared to the spatial memory task. Additionally, experts demonstrated neurological efficiency in the DLPFC, left ventral lateral prefrontal lobe (L-VLPFC), and right orbitofrontal area (R-OFA), exhibiting significantly lower cerebral blood oxygen activation than novices in both task conditions.

## Discussion

### Behavioral results

The investigation revealed that individuals within the high-experience-level expert group exhibited consistently strong behavioral performance, characterized by rapid reaction times in spatial memory tasks and high accuracy in mental rotation tasks. In the realm of orienteering practice or competition, athletes engage in continuous recognition, memory retrieval of map information, spatial location calibration, and self-centered or imaginative rotation of target objects^40^. These cognitive processes enable them to swiftly match real-world scenes, underscoring the pivotal role of spatial memory and mental rotation abilities in orienteering^41^. Previous studies have suggested that athletes tend to demonstrate heightened accuracy and faster reaction times in mental rotation tasks^42,43^. According to skill acquisition theory^44^, prolonged specialized practice optimizes the structure and sequential regularization of processing variables, enhancing decision-making efficiency. Throughout the journey from novice to expert, cognitive load decreases, novice-specific practice duration is minimized, and task decision-making efficiency improves. Behavioral performance varied across different spatial tasks^45,46^, with spatial memory tasks consistently outperforming mental rotation tasks. Once again, the cognitive advantage of expert groups in non-specific tasks was corroborated.

The findings of this study indicated that spatial memory performance surpassed that of mental rotation in the low experience-level novice group, whereas mental rotation performance in the high-experience-level expert group outshone that of novices. These distinctions may be attributed to the influence of motor experience. Mental rotation involves an individual’s ability to perform representational and rotational operations in the brain, whereas spatial memory encompasses the perception, storage, and reproduction of spatial information, reflecting differences in the spatial cognitive processing components of the two tasks. In essence, spatial memory is pertinent to daily life and learning, easier to consolidate and deepen, while mental rotation necessitates specialized practice (in sports, mathematics, architecture, aerospace) for acquisition^47^. Motor experience likely enhances spatial memory performance by training individuals to use and integrate spatial information more effectively. Novice individuals, with limited training time and experience, exhibit cognitive decision-making processes biased toward the spatial memory domain. In contrast, expert individuals, after years of orienteering-specific training, employ specific spatial calibration strategies, effectively honing their mental rotation abilities. Orienteering athletes, frequently navigating complex environments^48^, may derive their superior performance in spatial memory tasks from the demands of their sport.

#### fNIRS results

The results from fNIRS analyses demonstrated elevated oxyhemoglobin levels in the dorsolateral prefrontal lobe (DLPFC) during the mental rotation task, a critical brain region implicated in inhibiting nonadaptive behaviors and facilitating appropriate behaviors. This effect was observed in both the highly experienced expert group and the less experienced novice group^49^, suggesting the involvement of the mental rotation task in spatial cognition and visual processing regions^50^. Consequently, an increased activation of the DLPFC during mental rotation tasks is a reasonable observation. The role of the DLPFC extends beyond mental rotation tasks, as it is instrumental in recruiting additional cognitive resources and establishing functional connections with networks in other brain regions. These networks, collectively known as “cognitive control networks” or “prefrontal-lateral networks,” play a crucial role in cognitive control, working memory, and visual processing, encompassing higher cognitive functions such as decision-making. When confronted with intricate cognitive tasks, the DLPFC and cognitive control networks are engaged to coordinate and manage activities across different brain regions, facilitating effective task completion.

The study further revealed that the blood oxygen activation in the DLPFC, left ventral lateral prefrontal lobe (L-VLPFC), and right orbitofrontal area (R-OFA) of highly experienced expert athletes was significantly lower than that of novices across both task conditions. This finding implies that the prolonged specialization of highly experienced expert athletes enhances motor experience, thereby improving the efficiency of brain-related cortex in spatial task processing^51^. Supporting the notion that motor experience and individual skill level enhancements can automate cognitive tasks, specific brain regions are selectively utilized for task-specific activities^52^. Additionally, a notable positive correlation was observed between prefrontal activation of the DLPFC and task difficulty, indicating its role as a reliable predictor for allocating task-related attentional resources^53^. Novices in the low-experience group necessitate mobilizing more cognitive resources dedicated to extracting visual information, resulting in heightened activation of relevant brain regions. Conversely, the expert group, enriched with specialized task experience, demonstrates more automatic, fast, and efficient cognitive processing, forming intuitive thinking and thereby weakening the associated brain activation^54^. This confirms that long-term specialized training leads to reduced prefrontal cortex activity in relevant tasks, indicating neuroefficacy^55,56^.

Experts showcased a cognitive advantage characterized by enhanced perceptual pattern recognition and representation, underscoring the pivotal role of this brain region. Furthermore, the current study’s results revealed substantial differences in cerebral blood oxygenation activation in the left ventrolateral prefrontal cortex (L-VLPFC) between highly experienced experts and less experienced novices across both task conditions. This finding deviates from previous observations indicating that cerebral hemispheric lateralization typically favors distinct aspects of response inhibition and working memory. Traditionally, left prefrontal cortex (PFC) activation is associated with top-down control of attentional tasks and verbal working memory, while right PFC activation is linked to visuospatial tasks. The disparity in our results may be attributed to prolonged orientation-specific practice and experience. Specialized athletes, through rational allocation of neural resources, excel in concentrating on core tasks, minimizing interference, and ensuring task accuracy. Conversely, non-athletes might need to invest more cognitive effort in task completion. This interpretation aligns with earlier orienteering-related studies wherein the ventrolateral prefrontal cortex (VLPFC) played a role in map-aware spatial cognition tasks. Blood oxygen concentration was notably lower in professional athletes compared to beginners, emphasizing the concentration and focus facilitated by long-term orienteering training. This heightened focus, coupled with task difficulty, contributed to enhanced blood oxygen concentration^57^. Studies investigating spatial perception also revealed that activation of the left VLPFC was significantly higher in novices compared to experts in real-world scenarios^58^.

## Materials and methods

### Experimental subjects

A total of 49 subjects were recruited for this experiment starting in August 2023 and were categorized into a high-experience expert group and a low-experience novice group based on their level of orienteering experience. The high-experience-expert group comprised seventeen individuals (10 males, 7 females, average age 21.5 ± 1.50), boasting an average training duration of 8.7 ± 2.75 years. Notably, all members of this group had achieved top positions in either the National Orienteering Championships or the National Orienteering Elite Competition, solidifying their status as national-level athletes.

On the other hand, the low-experience-novice group consisted of thirty-two participants (16 males, 16 females, average age 20.47 ± 0.99), with an average training duration of 2±0.48 years. This group was comprised of members from the varsity orienteering team of a university. Selection criteria for all subjects included normal or corrected vision, right-handedness, absence of past medical history, traumatic brain injuries, mental health issues, or cardiorespiratory diseases. Additionally, participants were required to be proficient in computer keyboard operation and have no prior involvement in similar experiments. The day before the experiment, participants received comprehensive information regarding the experiment, including the schedule and relevant precautions, and were instructed to ensure adequate sleep and maintain cleanliness. Informed consent was obtained from all participants before the experiment, and the study protocol .The experiment was conducted with the informed consent of all participants and in strict accordance with the Declaration of Helsinki. And We received approval and supervision from the Academic and Ethics Committee of Shaanxi Normal University (SNNU20230318).

#### Experimental design

The experiment aimed to investigate variations in spatial cognitive abilities and prefrontal cortex activation patterns among orienteers with distinct experience levels, particularly in non-specialized scenarios. Two well-established cognitive psychology task paradigms, namely spatial memory and mental rotation were employed^58^. Motor experience was treated as a between-group variable, while different spatial cognition tasks served as a within-group variable.

Throughout the testing session, participants were seated in front of a computer monitor and instructed to minimize head movements while completing the tasks. The “E-prime 3.0” software automatically recorded data on correct rates and reaction times. Simultaneously, cerebral oxygenation data were gathered using the fNIRS device. This comprehensive experimental design allowed for a nuanced exploration of the interplay between motor experience, spatial cognitive tasks, and prefrontal cortex activation characteristics in orienteers.

#### Experimental equipment and stimulus materials

The fNIRS equipment utilized in the experiment was a portable functional near-infrared spectroscopic imager (LIGHTNIRS system), manufactured by Shimadzu, Japan. This device employed three different wavelengths of near-infrared light (780 nm, 805 nm, and 830 nm) and operated at a sampling rate of 13.33 Hz. It effectively monitored cerebral oxygenated hemoglobin (HbO2), deoxygenated hemoglobin (HbR), and Total-Hb signals. Given its heightened sensitivity to changes in local blood flow in the brain compared to HbR, HbO2 was selected as the indicator reflecting neural activation levels in this study^59,60^.

The photoplethysmographic probes were strategically positioned in the prefrontal lobe (PFC), with the lowest probe placed along the Fp1-Fp2 line, using the international 10-20 localization system as a reference^61^. Employing the PFC template provided by the system, a multichannel layout consisting of 2 $\times$8 photoplethysmographic probes (8 transmitting and 8 receiving poles) was created, totaling 22 channels. Each pair of similar photoplethysmographic poles were evenly spaced in a row, with a 3 cm distance between neighboring poles.

**Figure 4:**
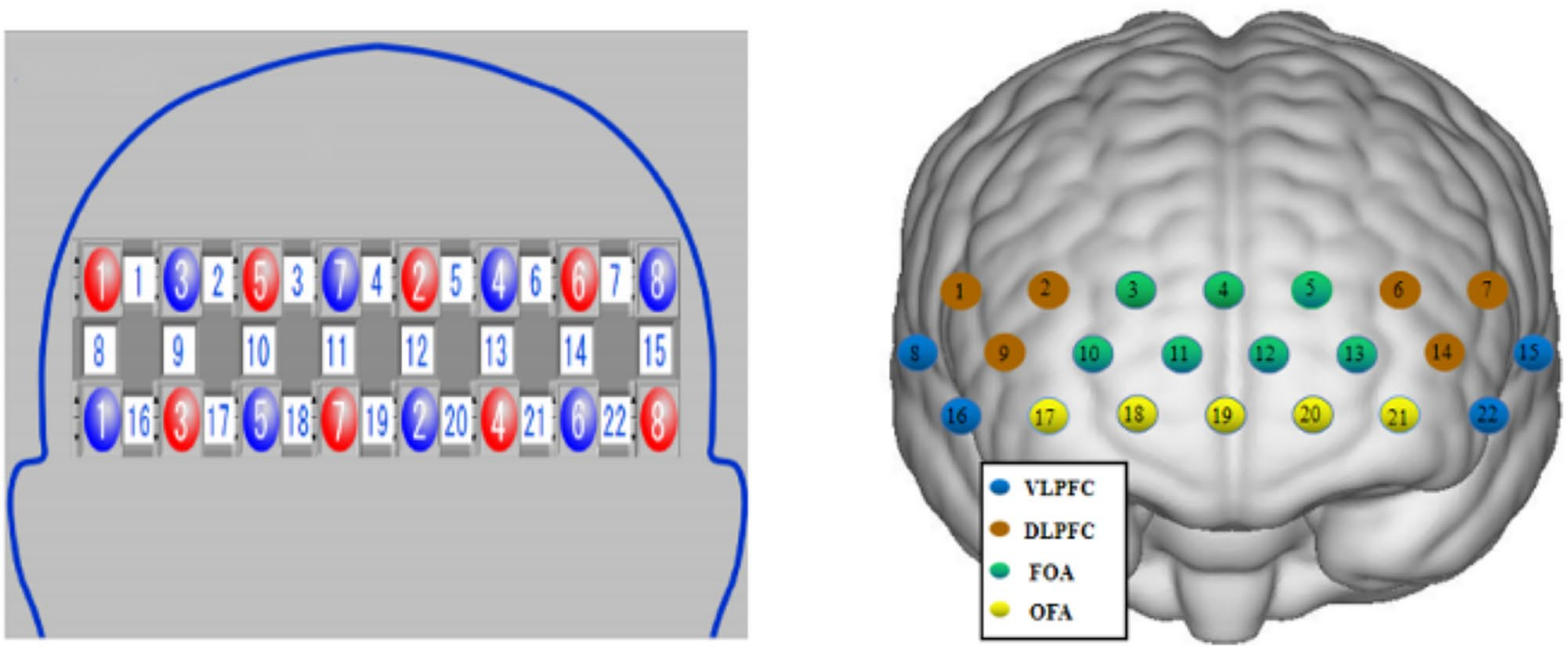
fNIRS channel layout and calibration brain region information (Note: red indicates emitter-signal source, blue indicates detector-detector, and numbers indicate -channel).

The 3D digitizer (FASTRAK system) was utilized for accurate optode positioning, and the spatial probabilistic alignment method of the NIRS_SPM software determined the MNI position coordinates for each channel. Subsequently, these coordinates were matched with the corresponding brain regions in the adult Brodmann area atlas. The calibration details are visually presented in Fig. 4 and comprehensively outlined in Table 2.

**Table 2:** Correspondence between the channel layout of the portable fNIRS device and the regions of interest of the brain (Note: Ch stands for Channel).

Brain region	Corresponding channel
Right dorsolateral prefrontal lobe (R-DLPFC)	Ch1, Ch2, Ch9
Left dorsolateral prefrontal (L-DLPFC)	Ch6, Ch7, Ch14
Right frontal pole area (R-FOA)	Ch3, Ch4, Ch10, Ch11
Left frontal pole area(L-FOA)	Ch4, Ch5, Ch12, Ch13
Right ventral lateral prefrontal (R-VLPFC)	Ch8, Ch16
Left ventral lateral prefrontal lobe (L-VLPFC)	Ch15, Ch22
Right orbitofrontal area(R-OFA)	Ch17, Ch18, Ch19
Left orbitofrontal area (L-OFA)	Ch19, Ch20, Ch21

All stimuli were displayed on a Dell laptop computer featuring a resolution of 1920 $\times$ 1080 and a refresh rate of 144 Hz. The experimental task sequences were executed using the neuropsychological programming software “E-prime 3.0.” Task stimuli were meticulously designed using specialized mapping software, grounded in established classical research paradigms.

**Figure 5:**
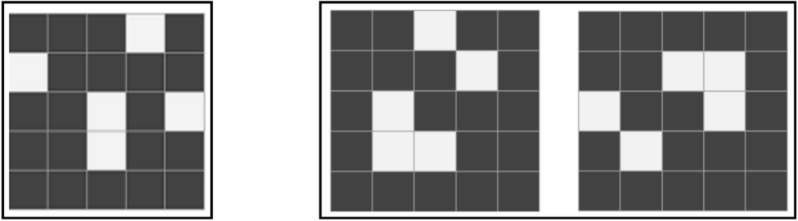
Experimental materials (Note:left: spatial memory;right:mental rotation).

The spatial memory task and the mental rotation task utilized stimulus materials in the form of a 5 $\times$ 5 matrix. Within this matrix, 4-5 small black squares were randomly positioned, conforming to the task requirements imposed on the subjects. A visual representation of the task stimuli is illustrated in Fig. 5

#### Experimental procedure

Prior to the commencement of the experiment, participants refrained from engaging in strenuous exercise to ensure clear mental focus. Upon entering the laboratory, subjects familiarized themselves with the environment, underwent physical and mental relaxation, and received thorough explanations regarding relevant precautions. Demographic information, including gender, age, training duration, and exercise level, was recorded, and subjects were briefed on experiment-specific precautions.

Subsequently, the experimental staff fitted each subject with the fNIRS photopolar cap, ensuring proper adjustment and conducting precise positioning calibration. Following the confirmation of normal light emission and reception for each channel, and after stabilization of signals, a zero reset was executed, marking the initiation of measurements. Each subject underwent individual testing.

Seated 80 cm in front of the display, subjects adjusted to a comfortable position, placing their fingers on the test keyboard to mitigate the influence of extraneous factors on experimental results. Prior to the formal experiment, a pre-experiment involving five orienteers was conducted. Based on the results, the initially designed 6-second reaction time for the Mental Rotation task was adjusted to 5 s, while other procedures remained unchanged. None of the participants in the pre-experiment participated in the formal experiment.

Experiments for the low-experience-novice group were conducted in the Sports Learning Science Laboratory of Shaanxi Normal University, whereas those for the high-experience-level expert group took place in the Athletes’ Office of Tianjin University of Finance and Economics. Behavioral data and prefrontal oxygenated hemoglobin concentration data were meticulously collected, providing insights into the spatial cognitive processing characteristics of orienteering athletes with varying experience levels in non-specialized scenarios.

The experiment comprised two distinct phases:a practice phase and the formal experiment. Both trajectories followed identical procedures. Guidance was provided, and subjects were instructed to familiarize themselves with the task content and operational processes. Upon pressing the space bar, a red “+” gaze point appeared at the center of the white background (complete computer display) for 1000 ms, capturing subjects’ attention. Subsequently, a task stimulus was presented, requiring subjects to make a judgment by pressing the corresponding key. Failure to make a judgment within the specified time resulted in the automatic progression to the next round. Feedback, indicating “correct,” “wrong,” or “no response,” was displayed at the center of the screen based on subjects’ judgments in the practice phase.It is crucial to note that stimuli used in the practice phase differed from those employed in the formal experiment. The primary objective of the practice phase was to acquaint subjects with the experimental procedure, with no data collection during this period. To cumulatively induce robust blood oxygenation effects and identify relevant activated brain regions, a block design was adopted.

#### Spatial memory task

**Figure 6:**
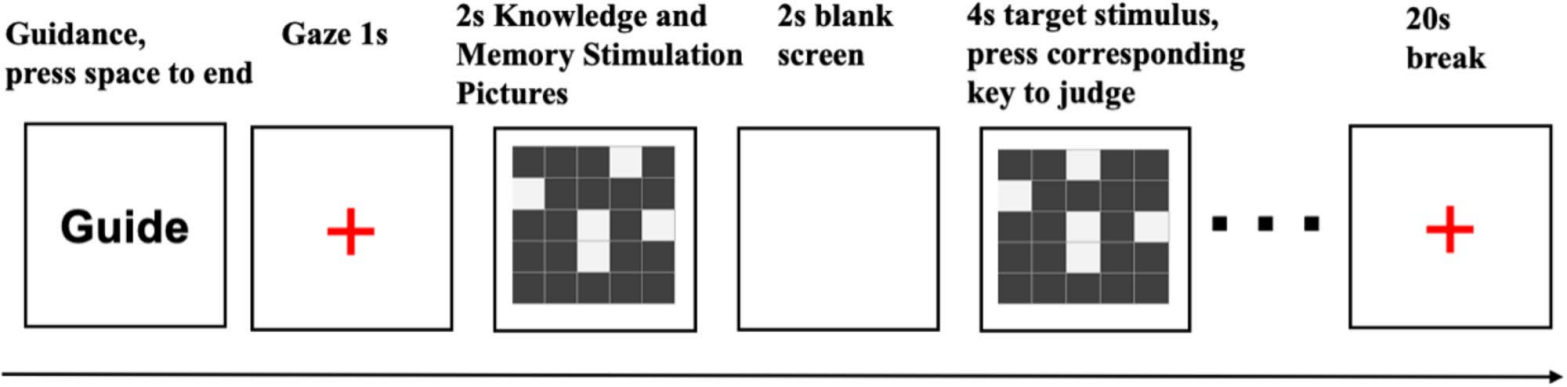
Flow chart of spatial memory task.

The instruction was presented on the complete computer screen, prompting subjects to familiarize themselves with the task content and operational processes. Upon pressing the space bar, a red “+” gaze point appeared for 1000 ms. Subsequently, a stimulus picture was presented for 2000 ms, requiring subjects to memorize the exact position of a white small square within the matrix. Following a 1000 ms empty screen interval, a target stimulus picture was presented for 2000 ms. Subjects then performed a keystroke response to determine the congruency between the target stimulus and the memorized stimulus. If congruent, the “f” key was pressed; if not, the “j” key was pressed. Feedback on correctness and response time was provided at the conclusion of each keystroke. Rest intervals were initiated at the end of the exercise, during which resting-state cerebral blood flow was collected. The formal experiment mirrored the practice phase, with subjects making judgments without feedback, and stimuli presented in a loop until the completion of the test. Each block comprised 4 trials, with a total of 6 blocks and a 20-s rest interval between each block. In total, 24 trials were conducted, with the program automatically recording subjects’ task response time, correctness, and cerebral blood oxygenation data (Fig. 6 for the experimental flow).

#### Mental rotation task

The Mental Rotation task commenced with the presentation of instructions on a complete computer screen. Subjects were instructed to familiarize themselves with the task content and operational processes before initiating the exercise by pressing the space bar. A red “+” gaze point appeared for 1000 ms, succeeded by a stimulus picture lasting 5000ms. Participants were tasked with determining whether the right graphic was a rotated version of the left graphic. If affirmative, the “f” key was pressed; otherwise, the “j” key was pressed. Feedback on correctness and response time was provided at the conclusion of each key press. Following the exercise, a break was implemented to collect resting cerebral blood flow.

**Figure 7:**
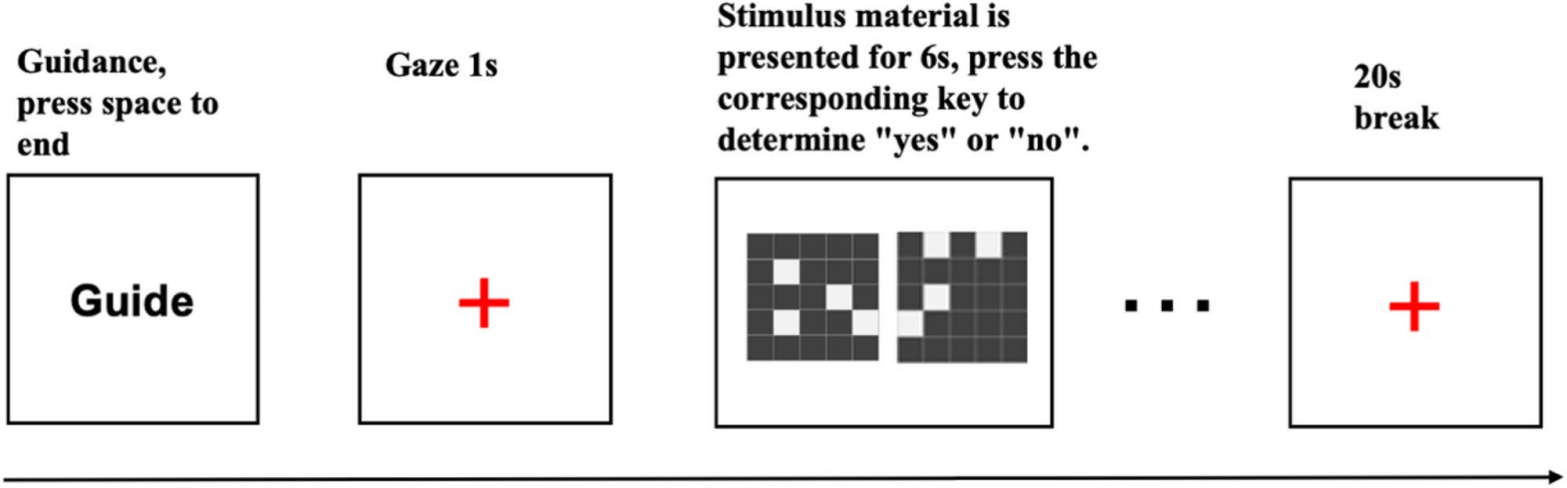
Flowchart of the mental rotation task.

The formal experiment mirrored the practice phase, with subjects making judgments without feedback, and stimuli presented in a loop until the conclusion of the test. Each block comprised 4 trials, totaling 6 blocks, with a 20-s rest interval between each block. In total, 24 trials were conducted, and the program automatically recorded subjects’ task response time, correctness, and cerebral blood oxygenation data (Fig. 7 for the experimental flow).

#### Data collection and analysis

##### Behavioral data analysis

The measurement data underwent normal distribution testing using SPSS 26.0, where a value exceeding the 0.05 threshold indicated adherence to a normal distribution. A 2 (group: high experience-level expert group, low experience-level novice group) $\times$ 2 (task type: spatial memory, mental rotation) two-factor repeated-measures ANOVA was employed to statistically analyze the correctness rate of spatial cognition tasks in non-specialized scenarios. This analysis aimed to discern the main and interaction effects of movement experience on different spatial cognition tasks. In instances where the interaction was significant, further analysis of simple effects was conducted. Throughout the analysis, violations of the assumption of sphericity were corrected using Greenhouse’s method, with significance set at p < 0.05. Visualization of the results was accomplished using GraphPad Prism 8.0. Given the variance in task design duration, response times were not subjected to inter-task comparisons. Instead, independent samples t-tests were utilized to explore group differences in response times across tasks.

#### fNIRS data analysis

Throughout the testing process, meticulous record-keeping of each subject’s coding and channels with weak signal connections was conducted. Adjustments or eliminations were made during the raw data processing. The software accompanying the fNIRS device facilitated the decoding of raw data. The NIRS_SPM software, operating on the Matlab (R2013b) platform, converted light intensity data to blood oxygenation data using the modified Beer-Lambert law. Subsequent data preprocessing involved outlier elimination and signal-to-noise ratio enhancement. This rendered the overall filtered signals amenable to subsequent analyses, encompassing MNI coordinates alignment, construction of the design matrix based on the general linear model (GLM), low-pass filtering using the hemodynamic response function (HRF) with a time derivative, and high-pass filtering employing the discrete cosine transform (DCT) de-trending algorithm. Evaluation of Beta values under task conditions served as an indicator of channel activation, with a higher Beta denoting greater brain region activation. Elevated Beta values reflected heightened activation in the corresponding brain area. fig Cerebral oximetry data underwent normal distribution testing using SPSS 26.0, with the Shapiro-Wilk test indicating data adherence to a normal distribution (p > 0.05). A two-factor repeated-measures ANOVA was then conducted for 2 (motor level: expert, novice) $\times$ 2 (task type: spatial memory, mental rotation). Corrections for violations of sphericity assumptions utilized the Greenhouse method. Significance was set at p < 0.05, and a more stringent threshold of p < 0.01 was also applied. 3D brain modeling heat mapping was executed using the BrainNet Viewer toolkit under Matlab software^62^.

LaTeX formats citations and references automatically using the bibliography records in your .bib file, which you can edit via the project menu. Use the cite command for an inline citation, e.g.^1^.

### Supplementary Information

Supplementary Information.

## Data Availability

Data completely open, free to download, in a public database (10.6084/m9.Figshare. 25572162), which can be found. The data in Table 1 and Fig. 1 are derived from the Non-specific (Novice) and Non-specific (Expert) data in the Supplementary Material raw data, comparing the behavioral performance of the High-Experience Expert Group with that of the Low-Experience Novice Group.Figures II and III on fnirs are derived from fNIRS (Novice) and fNIRS (Expert) data from the original data in the Supplementary Material, comparing cerebral oximetry characteristics of the highly experienced expert group with those of the inexperienced novice group.
